# Effects of Dietary Polyphenols from Olive Mill Waste Waters on Inflammatory and Apoptotic Effectors in Rabbit Ovary

**DOI:** 10.3390/ani11061727

**Published:** 2021-06-09

**Authors:** Margherita Maranesi, Cecilia Dall’Aglio, Gabriele Acuti, Katia Cappelli, Massimo Trabalza Marinucci, Roberta Galarini, Chiara Suvieri, Massimo Zerani

**Affiliations:** 1 Dipartimento di Medicina Veterinaria, Università di Perugia, via San Costanzo 4, 06126 Perugia, Italy; margherita.maranesi@unipg.it (M.M.); cecilia.dallaglio@unipg.it (C.D.); massimo.trabalzamarinucci@unipg.it (M.T.M.); massimo.zerani@unipg.it (M.Z.); 2 Istituto Zooprofilattico Sperimentale dell’Umbria e delle Marche “Togo Rosati”, via Gaetano Salvemini 1, 06126 Perugia, Italy; r.galarini@izsum.it; 3 Dipartimento di Medicina e Chirurgia, Sezione di Farmacologia, Università di Perugia, piazzale Severi 1, 06132 Perugia, Italy; chiara.suvieri@unipg.it

**Keywords:** apoptosis, BAX, COX2, IL1B, inflammation, olive waste, ovary, polyphenols, rabbit, TNFA

## Abstract

**Simple Summary:**

Circular economy strategies applied in the agro-food supply chain have recently been focused on new techniques for using agri-food by-products in animal nutrition. Rabbits efficiently convert plant proteins into foods rich in animal protein, therefore its meat, which contains numerous compounds, is potentially beneficial to human health and represents an excellent example of functional food. The residues obtained from the olive oil extraction process are a key source of phenolic compounds. In recent years, polyphenols have been used as dietary supplements to enhance animal health, welfare and performance, and to obtain functional foods of animal origin. Polyphenols enhance primary and total follicle number, and influence the inflammatory and apoptotic process by controlling and inhibiting pro-inflammatory cytokines (e.g., cyclooxygenase-2, interleukin-1beta, and tumor necrosis factor-alpha), and apoptotic factors (e.g., BCL2-associated X protein). Many health benefits of polyphenol consumption have not yet been determined, including the molecular and cellular mechanisms responsible for these actions at ovarian level. This paper highlights that dietary polyphenols, obtained from the olive oil industry, inhibit inflammatory and apoptotic activities in rabbit ovary, by modulating gene and protein expressions of cyclooxygenase-2 and BCL2-associated X protein, thus suggesting a direct involvement of these dietary compounds in mammalian reproduction.

**Abstract:**

The aim of this study was to evaluate the effect of dietary polyphenols on the expression of the effectors involved in inflammation and apoptosis in rabbit ovary. New Zealand White female rabbits were fed a basal control diet (CTR), or the same diet supplemented with a polyphenolic concentrate (POL, 282.4 mg/kg) obtained from olive mill waste waters. The follicle counts and the relative mRNA (RT-qPCR) and protein (immunohistochemistry) expression of the effectors involved in inflammation (cyclooxygenase-2; interleukin-1beta; tumor necrosis factor-alpha, TNFA) and apoptosis (BCL2-associated X protein, BAX), detected in the ovaries of both groups, were examined. The POL diet increased the primary and total follicles number. *Cyclooxygenase-2* gene expression was higher (*p* < 0.05) in the POL group than in the CTR group, whereas *BAX* was lower (*p* < 0.05) in POL than CTR. Immunohistochemistry revealed the presence of all the proteins examined, with weaker (*p* < 0.05) COX2 and BAX signals in POL. No differences between the CTR and POL groups were observed for IL1B and TNFA gene and protein expression. These preliminary findings show that dietary polyphenols modulate inflammatory and apoptotic activities in rabbit ovary, regulating cyclooxygenase-2 and BAX expression, thus suggesting a functional involvement of these dietary compounds in mammalian reproduction.

## 1. Introduction

Since the turn of the millennium many papers have been published on the efficient recycling of the agricultural by-products, due to their relevant significant environmental impact [1]. A circular economy is a system that is restorative or regenerative by intention and design, it replaces the end-of-life concept with restoration, shifts towards the use of renewable energy, eliminates the use of toxic chemicals, which impair reuse and return to the biosphere, and aims to eliminate waste [2]. In this context, circular economy strategies applied in the agro-food chain have recently started to focus on new techniques for using agri-food by-products in animal nutrition [3,4,5,6]. After poultry, rabbits are the most efficient converters of proteins contained in cellulose-rich plants into food with high-value animal protein, and are also a source of protein that is fairly simple to produce in rural communities and economically less-developed countries [7,8]. Due to the current societal focus on sustainability and avoidance of food-feed competition, nutrient-rich functional foods high in polyphenols, such as rabbit meat, prove to be particularly interesting [7]. The residues derived from the olive oil extraction process are a key source of phenolic compounds, such as hydroxytyrosol (3,4-DHPEA), tyrosol (p-HPEA), verbascoside (derived from hydroxycinnamic acid), caffeic acid, p-coumaric acid and oleuropein [9,10]. These bioactive molecules are structurally diverse natural compounds, whose common occurrence in plants renders them intrinsic dietary components [9]. In recent years, polyphenols have been used as dietary supplements to enhance animal health, welfare, and performance, and to produce functional foods of animal origin [4,11,12,13,14]. They are metabolites that have modulatory effects on physiological functions, including inflammation, apoptosis and metabolism [15,16,17]. Polyphenols modulate the inflammatory process by controlling and inhibiting the production of pro-inflammatory cytokines, e.g., interleukin-1beta (IL1B), IL-6, IL-8 and tumor necrosis factor alpha (TNFA) and cyclooxygenase-2 (COX2) enzyme [18]. Many studies have found that these dietary compounds have both pro-apoptotic and anti-apoptotic effects [19,20,21,22]. Dietary polyphenols have proven to influence fertility, sexual development and fetal health [16]. Interesting reviews, addressing the effects of polyphenolic compounds on ovarian and reproductive functions, have recently been published [23,24]. In this context, Balazi et al. [25] suggested that dietary polyphenols can reduce the ovarian functions and fecundity of rabbit does.

After many years of a slump in rabbit meat consumption and the low price of rabbit fur, the commercial rabbit sector is facing fundamental challenges, which will shape its future and threaten its sustainability [26]. Gaining new insights on the reproductive physiology of this animal species may help breeders to improve rabbit meat production efficiency.

Due to their size, rabbits are easy animals to breed as they do not require large amounts of space. Moreover, rabbit is a reflex ovulating species, which maintains peripheral concentrations of estradiol-17beta and progesterone at basal levels in unmated females, in contrast to spontaneous ovulators [27]. For this reason, the rabbit is a suitable species for investigating the effects of experimental substances on the ovary, given the absence of variations in gonadal steroids [27].

Ovulation is a process accompanied by acute inflammatory events that are driven by COX2 expression [28], which is a rate-limiting enzyme in prostaglandin PGE2 synthesis [29]. In the ovary, this prostaglandin regulates angiogenesis, blood flow, immune cell function and tissue remodeling associated with cumulus expansion, follicle wall proteolysis and the formation of the corpus luteum [28]. More importantly, COX2-induced PGE2 is an inflammatory mediator that, combined with pro-inflammatory cytokines such as IL1 and TNFA, induces a similar inflammation process in the ovulating ovary [30,31]. In the absence of an ovulatory gonadotropic trigger, IL1B has been identified as a cytokine that induces ovulation, oocyte maturation, and facilitates fertilization [32]. IL1 also modulates gonadotropin action on steroidogenesis through cAMP production [33]. In regards to TNFA, this molecule suppresses the responsiveness of the ovary to gonadotropins in small developing follicles, while it stimulates steroidogenesis in preovulatory follicles [34]. Several authors [35,36,37,38] have identified BCL2-associated X protein (BAX) in the rabbit ovary, as it regulates the cyclic nucleotide-dependent apoptotic process [37] and PGF2alpha-induced luteolysis [35].

In light of the above, the aim of the present study was to highlight the possible effects on follicle presence and on gene and protein expression of inflammatory (COX2, IL1B, TNFA) and apoptotic (BAX) effectors in the ovary of rabbits fed with polyphenols derived from the olive oil industry.

## 2. Materials and Methods

### 2.1. Reagents

The reagent for the isolation of total RNA (TRIzol) was purchased from Invitrogen (S. Giuliano Milanese, Milano, Italy). iSCRIPT cDNA and iQ SYBR Green SuperMix were purchased from Bio-Rad Laboratories (Hercules, CA, USA, 2009). RT-qPCR primers for *COX2*, *BAX*, *IL1B*, *TNFA* and *18S* were supplied by Sigma-Aldrich (St. Louis, MO, USA) as well as RNA storage solution (RNAlater). Reagents for immunohistochemistry (IHC) and Western blot (WB) are as follows: the primary antibodies mouse anti-BAX (sc-7480), mouse anti-COX2 (sc-376861) were supplied by Santa Cruz Biotechnology (Santa Cruz, CA, USA), as well as mouse anti-IL1B (sc-32294), mouse anti-TNFA (sc-130349) used for IHC; the mouse monoclonal anti-beta-tubulin antibody was from Sigma-Aldrich (St. Louis, MO, USA), the secondary biotinylated horse anti-mouse IgG antibody (BA-2000-1.5), the avidin–biotin complex (ABC Elite Kit, PK-6200) and the diaminobenzidine (DAB, SK-4100) were supplied by Vector Laboratories (Vector Labs, Burlingame, CA, USA). The Eukitt (03989) was purchased from Sigma-Aldrich (St. Louis, MO, USA). PageRuler Protein Ladder for WB was obtained from Fermentas (Burlington, Ontario, Canada). The horseradish peroxidase (HRP)-conjugated IgG secondary antibody, used for WB as the Restore Western blot stripping buffer, was obtained from Thermo Fisher Scientific (Rockford, IL, USA). Protran nitrocellulose membranes were purchased from Whatman (Dassel, Germany). Biomax films used to document immunocomplexes were acquired from Kodak Laboratories (Rochester, NY, USA). The enhanced chemiluminescence detection system for WB (Immobilon Western Chemiluminescent HRP Substrate, Sigma-Aldrich) was purchased from Millipore (Billerica, MA, USA). The bands were quantified using Quantity One software, version 4.6.3 (Bio-Rad Laboratories, Hercules, CA, USA, 2009).

### 2.2. Animals and Treatments

Eight New Zealand White female rabbits were weaned at 30 days of age and randomly assigned to two dietary groups balanced for live weight (617.14 ± 74.10 g). The animals were housed individually under controlled conditions of light (16 h light/8 h darkness), temperature (18 to 23 °C) and humidity (60 ± 5%). The rabbits were either fed with a commercial pelleted concentrate (control group, CTR; n = 4) or the same concentrate supplemented with an olive mill waste water (OW) product (Poli4Life^®^, Terni, Italy) to reach a total phenolic content of 282.4 mg/kg (POL group, n = 4). Experimental feeds were sampled three times (at the beginning, in the middle and at the end of the trial) following the Commission Regulation (EC) No. 691/2013 and analyzed in accordance with AOAC methods [39] and Maertens et al [40].

The composition of the commercial feed pellets is reported in Table S1. The polyphenol contents of the CTR and OW feeds (Table 1) were determined by applying liquid chromatography coupled with tandem mass spectrometry (LC-MS/MS) as previously described [12]. 

Fresh water was provided and rabbits did not show any clinical signs of illness throughout the entire trial. No mortalities were recorded. The rabbit does (average fasting weight: CTR 2263.57 ± 235.79 g, POL 2247.14 ± 137.38 g) were slaughtered in a private slaughterhouse at 90 days of age according to standard commercial procedures and welfare codes of practice.

After slaughter, the ovaries were promptly removed and thoroughly washed with saline [41]. Within minutes the ovarian specimens were processed for IHC [42] or were cut into appropriately sized pieces (approximately 50 mg) and placed in tubes containing RNAlater. When they were removed from the solution, the samples were frozen within 24 h at −80 °C so that they could be later evaluated for gene expression and WB [43].

### 2.3. Morphometric and Histological Analysis of Ovaries

The following information was acquired: length and width of each ovary [44]. Ovarian follicular count was also performed [45]. Sections (5 µm thick) were cut from each sample and mounted on standard glass slides. The sections were stained with hematoxylin-eosin. The identification, classification and count of primary, secondary and tertiary ovarian follicles were done as follows and as previously described [45,46]: primary follicles, single layer of cuboidal cells surrounding the oocyte; secondary follicles, more than one layer of granulosa cells with no visible antrum; tertiary follicles, presence of multiple layers of granulosa cells and visible antrum. The number of follicles, examined in one section, varied from 1 to 50/per section. All follicles were measured twice and only follicles with well-visible oocytes were measured using a light microscope Eclipse E800, (Nikon Corporation, Tokyo, Japan) connected to a digital camera (Dxm 1200 Nikon digital camera).

### 2.4. Immunohistochemistry

The immunohistochemical procedure was carried out as previously described [42]. After 24 h of fixation by immersion in 4% formaldehyde in phosphate-buffered saline solution (pH 7.4, PBS), the samples were dehydrated through a series of graded ethanol baths, embedded in paraffin wax and cut into 5 µm serial sections. The tissue sections were then deparaffinized in xylene, rehydrated in graded ethanol and finally rinsed in distilled water. The sections were then immersed in 3% H_2_O_2_ in buffer for 10 min to quench the endogenous peroxidase activity and rinsed in PBS. Subsequent steps were performed at room temperature in a moist chamber in order to prevent the evaporation of reagents. After incubation with normal goat serum for 30 min, the serial sections were incubated overnight with all primary antibodies diluted 1:100 in PBS. The following day all of the sections, except for those incubated with IL1B, were rinsed in PBS and incubated with the secondary antibody (dilution 1/200) for 30 min and then after another rinse with PBS, the sections were incubated in avidin–biotin–peroxidase, diluted 1:50:1 in PBS, for further 30 min. Finally, all of the sections were incubated with diaminobenzidine solution for 5–10 min. The sections in which the primary antibodies were omitted and/or replaced with pre-immune mouse-globulin were used for the negative control of unspecific staining (Figure S1). All tissue analyses were carried out on randomly selected slides using a light microscope Eclipse E800, (Nikon Corporation, Tokyo, Japan) connected to a digital camera (Dxm 1200 Nikon digital camera, Nikon Corporation). The images were processed using an image analysis system (IAAS 2000 image analyzer, Delta Sistemi, Rome, Italy), that was calibrated by taking the background developed in sections incubated with non-immune serum as ‘zero’[47]. Data are expressed as average optical density.

### 2.5. Protein Analysis of BAX, COX2 by Western Blotting

The changes in expression of ovarian BAX and COX2 proteins were analyzed by WB in the ovary of rabbits of CTR and POL groups. For each rabbit, total ovarian proteins were extracted from the ovarian tissue, as previously described [35]. Briefly, the ovarian tissues were homogenized in 300 μL ice-cold RIPA buffer (PBS containing 1% Igepal CA-630, 0.5% sodium deoxycholate, and 0.1% SDS) containing a protease inhibitor (cOmplete™ Protease Inhibitor Cocktail, Roche, Basel, Switzerland) under agitation for 2 h at 4 °C. After incubation at 4 °C for 20 min, the homogenates were centrifuged at 12,000 g for 60 min at 4 °C. The protein concentrations of supernatants were measured using the protein assay kit with bovine serum albumin (BSA) as standard. Equivalent amounts of protein (50 μg) were separated by discontinuous 12% SDS-PAGE with 4% staking gel for 40 min at 200 V and 500 mA. Thereafter, proteins were transferred onto nitrocellulose membrane with Trans-Blot Turbo system (Bio-Rad Laboratories). The membrane was then blocked in Tris-buffered saline (TBS) containing 0.05% Tween 20, and 3% BSA. Immunoblotting was performed by sequential exposure to anti-BAX and anti-COX2 monoclonal antibodies (1:500) overnight at 4 °C. Membrane was then probed with HRP-labeled anti-mouse IgG antibody (1:10,000), respectively, for 60 min at room temperature under gentle agitation. All antibody incubations were performed in TBS containing 5% non-fat dried milk and 0.05% Tween 20. The immunocomplexes were detected by enhanced chemiluminescence according to the manufacturer’s protocol (Clarity Western ECL Substrate, Bio-Rad Laboratories). Blot images were acquired, and the intensities of the bands were quantified by densitometric analysis. After the blots were stripped, membranes were reproved with mouse anti-tubulin monoclonal antibody (1:1000) for two hours at room temperature. Values were expressed as arbitrary units of relative abundance of the specific proteins normalized with that of beta-tubulin used as loading control.

### 2.6. RNA Extraction and RT-qPCR

Total RNA was extracted from each rabbit ovary as previously described [48]. Five micrograms of total RNA was reverse transcribed in 20 µL of iSCRIPT cDNA using random hexamer according to the protocol provided by the manufacturer. Genomic DNA contamination was checked by developing an RT-qPCR assay without reverse transcriptase [49]. Serial experiments were carried out to optimize the quantitative reaction, efficiency and Ct values. The optimal 25 µL RT-qPCR reaction volume contained 12.5 µL of iQ SYBR Green SuperMix, 1 µL forward and reverse primers (stock concentration 10 µM) and 25 µL of water. The primers used [35,50,51] are listed in Table 2. All reagents were mixed as a master mix and distributed into a 96-well RT-qPCR plate before adding 2 µL of cDNA for each gene (diluted 10-fold with water). For every PCR run, negative reaction controls without reverse transcriptase in RT were included to determine that RNA was free of genomic DNA contamination. Samples amplification fidelity was also verified by agarose gel electrophoresis. RT-qPCR was performed on an iCycler iQ (Bio-Rad Laboratories) with an initial incubation at 95 °C for 1.5 min, followed by 40 cycles at 95 °C for 15 s, 53 °C for 30 s, during which fluorescence data were collected. The cycle threshold (CT) value was automatically computed for each trace. The *18S* Ct housekeeping gene was determined to normalize samples variations in the amount of starting cDNA. Standard curves were generated by plotting the Ct against the log cDNA standard dilution (1/5 dilution) in nuclease-free water. The slope of these graphs was used to determine reaction efficiency (Figure S2). Quantification of the standard curve was evaluated using iCycler system software (Bio-Rad Laboratories, Hercules, CA, USA) while mRNA gene expression was quantified with the 2^-ΔΔCt^ method [52]. The melting curve analysis, performed immediately after the RT-qPCR end cycle, was used to determine the specificity of each primer set. A melt curve protocol was performed by repeating 80 heating cycles for 10 s, from 55 °C with 0.5 °C increments, during which fluorescence data were collected.

### 2.7. Statistical Analysis

Data were examined using the Student’s *t*-test for parametric data (follicles count, RT-qPCR) and Kruskal–Wallis test for non-parametric (WB, average optical density). Differences were considered significant at *p* < 0.05. Equality of variances was checked by Levene’s test.

## 3. Results

### 3.1. Morphological and Histological Analysis of Ovaries

The morphometric evaluation of the ovaries did not reveal the influence of feeding with polyphenols on ovarian size (length: CTR 1.61 ± 0.08 cm, POL 1.62 ± 0.11 cm; width: CTR 0.63 ± 0.05 cm, POL 0.61 ± 0.12 cm).

**Figure 1:**
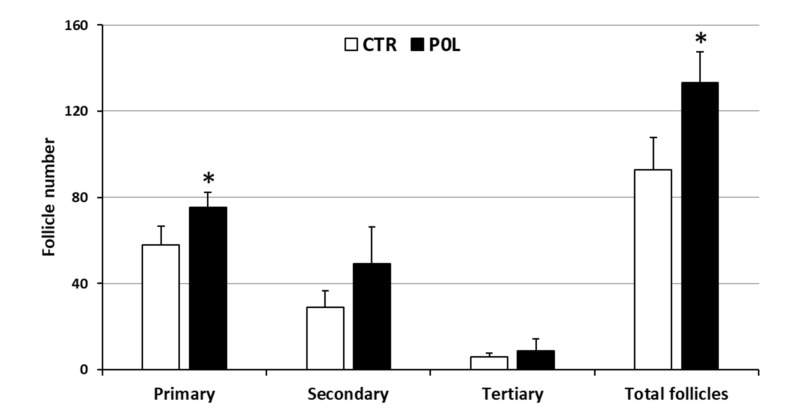
Primary, secondary and tertiary follicles counting in the ovary belonging to rabbits fed with a commercial pellet alone (CTR) or supplemented with an olive mill waste water product to reach a total concentration of 282.4 mg/kg of phenolic compounds (POL). Data represent the mean ± SD of eight replicates. Student’s *t*-test: * *p* < 0.05 CTR vs. POL (primary: *p* = 0.0217; secondary: *p* = 0.0717; tertiary: *p* = 0.3873; total follicles: *p* = 0.0083). Levene’s test: primary: *p* = 0.3571; secondary: *p* = 0.3480; tertiary: *p* = 0.5082; total follicles: *p* = 0.9401.

Polyphenols increased (*p* < 0.05) the number of primary and total follicles in the POL group, while the number of secondary and tertiary follicles were unaffected (Figure 1). 

### 3.2. Immunohistochemistry

**Figure 2:**
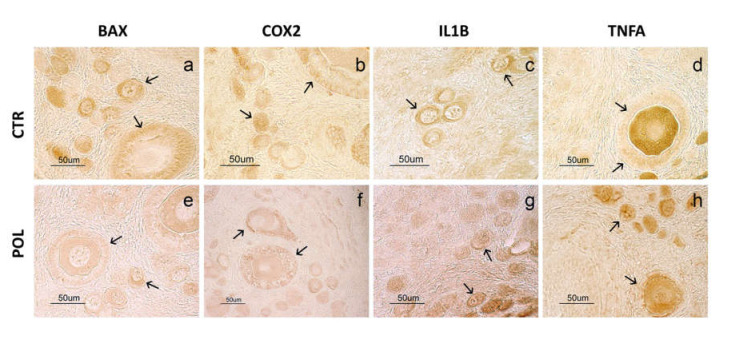

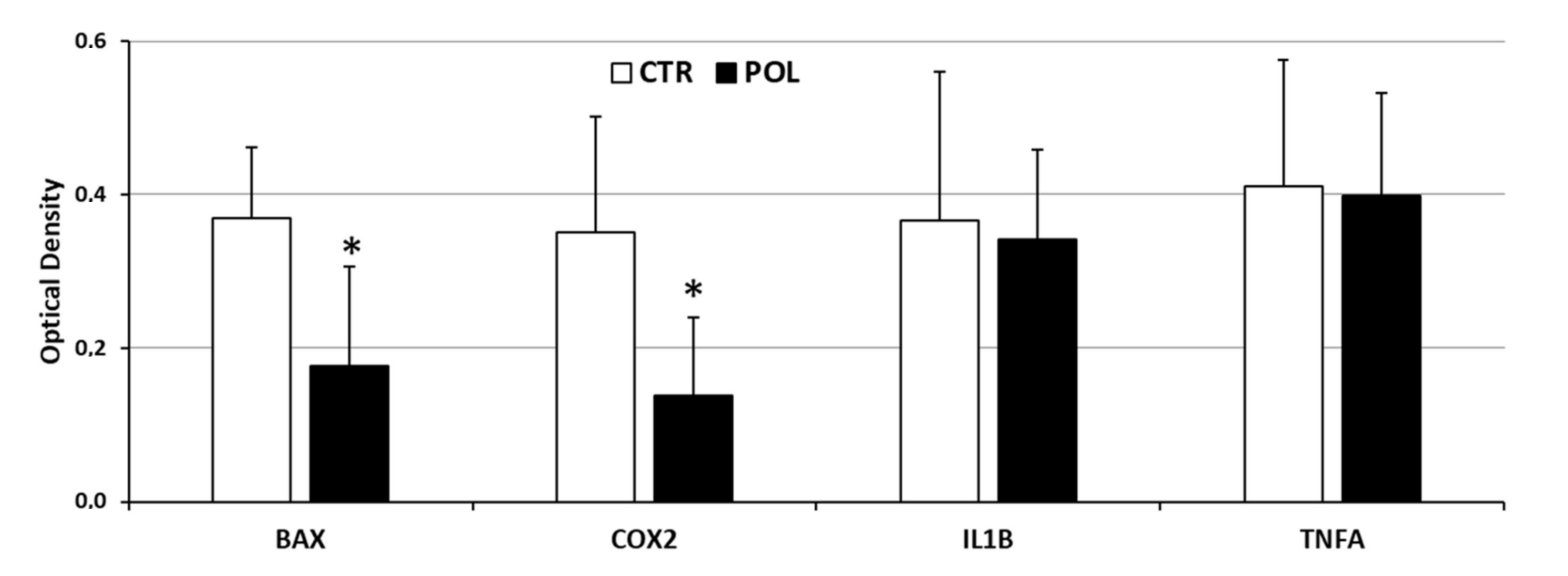
Immunohistochemistry in ovary tissue sections of rabbits fed with a commercial pellet alone (CTR) or supplemented with an olive mill waste water product to reach a total concentration of 282.4 mg/kg of phenolic compounds (POL). Upper panel: immunohistochemical reactivity for BAX (**a**,**e**), COX2 (**b**,**f**), IL1B (**c**,**g**), TNFA (**d**,**h**). DAB staining is evident in the cytoplasm of primordial, primary follicular cells (arrows) and some egg cells (asterisk) of both groups (for more details see the text). Lower panel: intensity of staining expressed as average optical density. Data represent the mean ± SD of 10 replicates. Kruskal–Wallis test: * *p* < 0.05 CTR vs. POL (BAX: *p* = 0.0018; COX2: *p* = 0.0027; IL1B: *p* = 0.7258; TNFA: *p* = 0.8509). Levene’s test: BAX: *p* = 0.4304; COX2: *p* = 0.5202; IL1B: *p* = 0.7963; TNFA: *p* = 0.3533.

The immunohistochemical studies revealed a positive immunoreaction (IR) for BAX, IL1B, COX2, and TNFA in some ovarian structures, with a similar distribution pattern (Figure 2). In particular, the positive IR for IL1B, BAX and TNFA was evident in the primordial and primary follicles, involving the cytoplasm of follicular and granulosa cells and the egg cell, while it was absent in the more developed follicles (Figure 2, upper panel; Figure S3). COX2 IR was evident in primordial, primary and secondary follicles, involving the cytoplasm of follicular and granulosa cells and the egg cell (Figure 2, upper panel). On comparing control and treated animals, POL treatment affected the positive IR for BAX and COX2 with a weaker signal (*p* < 0.05) than CTR animals, while the IR for IL1B and TNFA was the same for both groups of animals (Figure 2, lower panel).

### 3.3. Western Blotting

**Figure 3:**
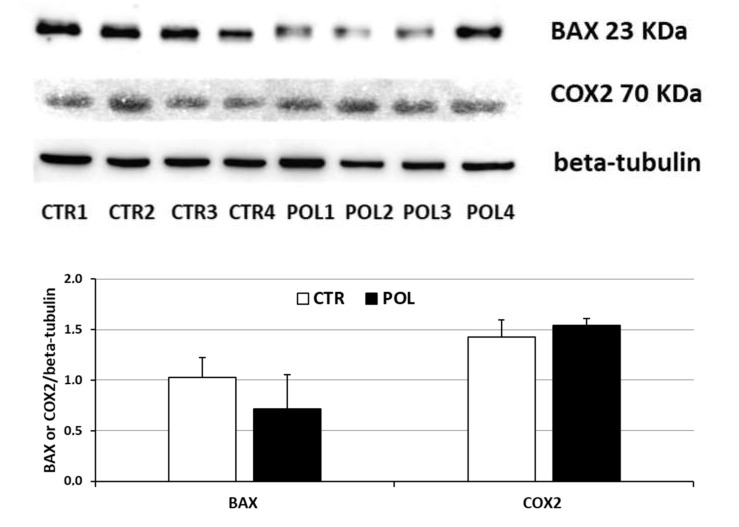
Upper panel: representative immunoblots of BAX, COX2 and beta-tubulin proteins in the ovary of rabbits fed with a commercial pellet alone (CTR) or supplemented with an olive mill waste water product (phenolic content of 282.4 mg/kg: POL); doublet bands at approximately 23 KDa were detected for BAX, a strong band at 70 KDa was detected for COX2. Lower panel: densitometric analyses of the BAX and COX2 blots are reported in arbitrary units relative to beta-tubulin. Data represent the mean ± SD of four replicates. Kruskal–Wallis test: BAX: *p* = 0.1611; COX2: *p* = 0.2382. Levene’s test: BAX: *p* = 0.7964; COX2: *p* = 0.3163.

Western blotting confirmed the specificity of the antisera used for BAX and COX2, showing in all the ovaries examined the typical doublet band at about 23 kDa for BAX and 70 kDa for COX2 (Figure 3 and Figure S4). No statistically significant differences were observed between the CTR and POL groups (Figure 3 and Figure S4).

### 3.4. RT-qPCR

**Figure 4:**
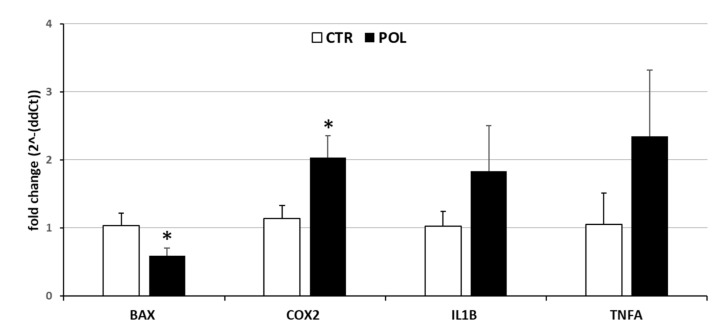
Gene expression levels of *BAX*, *COX2*, *IL1B*, and *TNFA* in ovary of rabbits fed with a commercial pellet alone (CTR, white bars) or supplemented with an olive mill waste water product (Poli4Life^®^) to reach a total concentration of 282.4 mg/kg of phenolic compounds (POL, black bars). Messenger RNA gene expression was calculated by the 2^-ΔΔCt^ method. Data represent the mean ± SD of four replicates. Student’s *t*-test: * *p* < 0.05 CTR vs. POL (*BAX*: *p* = 0.0064; *COX2*: *p* = 0.0027; *IL1B*: *p* = 0.0595; *TNFA*: *p* = 0.0526).

Higher expression levels of COX2 (*p* < 0.05) were observed for the POL group than the CTR group, while BAX expression was lower (*p* < 0.05) in POL compared to CTR (Figure 4). No differences were observed in IL1B and TNFA gene expression between the CTR and POL groups (Figure 4).

## 4. Discussion

This study reports new data on the effects of dietary polyphenols on inflammatory and apoptotic mechanisms in rabbit ovary.

In particular, our data suggest that this diet could enhance reproductive success of breeding species. In addition, regarding circular economy, reusing olive oil waste will be beneficial from an economic and environmental point of view.

Dietary polyphenols appear to affect ovarian function and lifespan with contrasting effects [16,25,53,54,55,56,57,58,59,60,61]. Tea polyphenols may inhibit the transition from primordial to developing follicles, reduce the number of dominant follicles in order to increase the reserve of germ cells, and inhibit oocyte apoptosis and follicle atresia during ovarian development in adult mice [54] and rats [55]. These findings show that polyphenols are beneficial plant compounds that can prolong ovarian activity. Since female mammals are born with a fixed number of oocytes that decline with age without the possibility of renewal [51,52], increasing the number of follicles available to adult females enhances their reproductive capacity. Our data agree with these authors, in fact the total number of follicles, and in particular that of the primary ones, was increased by the diet with OW. Similarly, other authors have demonstrated that polyphenols are capable of boosting bovine [56] and porcine [57] oocyte fertility rates. Conversely, other in vitro studies [55,56,57,58] suggest that polyphenols may negatively affect female reproductive health. Curcumin has been proven to induce mouse oocyte apoptosis, which causes a significant reduction in oocyte maturation rates [55]. Due to their estrogenic activity, flavonoids can disrupt the structural development of reproductive organs [56]. In pigs, tea polyphenols activate apoptosis [57], suppress proliferation, increase progesterone and estradiol release in cultured granulosa cells, and reduce oocyte fertility [61]. In regards to rabbits, Balazi et al. [25] suggested that dietary polyphenols can reduce rabbit doe’s viability, ovarian functions and fecundity, due to changes in ovarian cell apoptosis. More specifically, these authors reported that ovarian fragments, cultured with resveratrol or green tea polyphenols, reduced the accumulation of pro-apoptotic caspase 3 [25].

Our results are consistent with those of other authors who highlighted the positive effects of polyphenols on reproduction [53,54,55,56,57,58,59,60,61]. In fact, dietary polyphenols induced the downregulation of the BAX gene and protein expression, which promoted caspase 3 activation and therefore apoptosis. Interestingly, Sánchez-Rodríguez et al. [62] reported that treatment with polyphenols inhibits the activation of age-related apoptotic signaling in rat cochlea. In particular, the protein levels of pro-apoptotic Bcl2 in older rats were recovered after administration of polyphenols, while BAX protein levels decreased [63]. Moreover, walnut polyphenol extract proved to prevent organophosphate pesticide-induced toxicity to mice splenic lymphocytes in vitro, by reducing the expression of the apoptosis-associated protein BAX [63].

Ovulation, corpus luteum development, menstruation, implantation, and parturition are all inflammatory-like processes and, as such, physiological inflammatory responses are essential for reproductive success [16]. Polyphenols exert their anti-inflammatory effects through a range of molecular targets, which can be divided into the following two pathways: the arachidonic acid (AA)-dependent pathway and the AA-independent pathway [16]. In the former, phospholipase A2 releases AA present in cell membrane phospholipids, which is then converted into prostaglandins by COX1 and/or -2 [16]. COX2 is a pro-inflammatory mediator included in the AA-dependent pathway. Its activation leads to PGE2 synthesis and proinflammatory molecules such as IL1B and TNFA [64]. Several in vitro studies [64,65,66,67,68] have reported that polyphenols negatively affect the pro-inflammatory COX2 expression and activity. Chao et al. [65] reported that the polyphenol naringenin inhibited COX2 protein expression in macrophage and microglia cell lines under inflammatory stimulation induced by lipopolysaccharides. Avenanthramides, natural polyphenols obtained from oat sprouts, inhibited the enzymatic activity of pro-inflammatory COX2, and reduced PGE2 levels in CaCo-2 and Hep3B cancer cells [66]. Escin polyphenols in vitro quenched the gene expression of COX2 in human synoviocyte cells exposed to lipopolysaccharide stimulation [67]. Treatment with cloudy apple juice polyphenols reduced COX2 activity in the livers of injured animals [68], while the green tea polyphenol epigallocatechin-3-gallate significantly reduced *COX2* and *TNFA* mRNA expression levels in doxorubicin-induced inflammatory responses in human ovarian tissue [69]. Our protein expression data are in line with those obtained from the above-mentioned studies, thus confirming that polyphenols inhibit COX2 expression in rabbit ovary. Conversely, the gene expression data showed that OW polyphenols play an up-regulatory role, as they have a positive effect on *COX2* gene expression in rabbit ovary. Excluding possible technical problems in the experimental procedure, which we have not found, in order to explain the contrasting protein expression trends and gene expression data we can hypothesize that besides their effect on inflammatory processes, there are species-specific mechanisms of response to various polyphenols. Moreover, the high concentrations of phenols present in OW used to supplement commercial pelleted feeds (hydroxythirosol, tyrosol, and verbascoside) could modulate the inflammatory processes through different mechanisms, which may require the following post-transcriptional adjustments: gene up- and protein (mRNA transduction?) down-expression regulation. However, there is certainly a lack of scientific literature on the topic, as only three studies have reported the effects of hydroxythirosol, tyrosol and verbascoside on the ovary [70,71,72], which all investigated the protective effect on DNA in Chinese hamster ovary (CHO) cells. Verbascoside significantly reduced oxidative DNA damage by H_2_O_2_ in CHO cells, through non-enzymatic fast repair mechanisms [70]. Hwang et al. [71] evaluated the safety of *Acer tegmentosum*, which contains salidroside and tyrosol, and found that it did not significantly increase the number of chromosomal aberrations in CHO cells. *Leonurus sibiricus* plant extracts contain nine phenolic compounds, including verbascoside [72]; all tested extracts of *Leonurus sibiricus* proved to have protective and DNA repair stimulating effects in CHO cells exposed to H_2_O_2_ [73].

In their reviews, Ly et al. [16] and Gupta et al. [73] have reported extensively on the inhibitory effects of polyphenols on pro-inflammatory cytokines and chemokines, including TNFA and IL1B, in various tissues and species [16,73]. The most recent studies [74,75] also confirmed the inhibitory effects of polyphenols on TNFA and IL1B. The polyphenolic compounds present in *Sargassum horneri* extract down-regulated the inflammatory cytokines TNFA and IL1B in HaCaT keratinocytes [74]. *Camellia oleifera* oil has phenolic compounds that reduced in vitro TNFA and IL1B production in lipopolysaccharide-activated RAW 264.7 macrophage [75]. Conversely, the dietary polyphenols used in our study did not have any effects on the TNFA and IL1B genes and protein expression in rabbit ovary, species-specific differences and the low doses of polyphenols used in this study as suggested above.

## 5. Conclusions

In conclusion, although this study was based on a small sample, the data obtained suggest that dietary polyphenols play an important direct role in modulating ovarian activity in mammals. In particular, these dietary polyphenolic concentrates (dose: 282.4 mg/kg) influence inflammatory and apoptotic activities in rabbit ovary, by modulating gene and protein expressions of COX2 and BAX. Due to the effectiveness of these two mechanisms in regulating ovarian activity, this particular diet could enhance the reproductive success of breeding species. Moreover, there is also the positive aspect of reusing olive oil waste, which can bring both economic and environmental benefits, and accelerate our transition towards a more circular economy.

## Figures and Tables

**Table 1:** Total content of polyphenols (µg/g) of the two experimental concentrates used during the trial.

	Concentrate		Method LOD (µg/g)
	CTR ^a^	POL ^b^	
Hydroxytyrosol	0.4	182.0	0.05
Tyrosol	2.6	30.0	0.25
Verbascoside	2.5	70.0	0.05
Pinoresinol	0.3	0.4	0.05
Total polyphenols	5.8	282.4	

^a^ Commercial pelleted concentrate. ^b^ Commercial pelleted concentrate supplemented with Poli4Life^®^ (olive mill waste water product).

**Table 2:** Primers used for gene quantification by RT-qPCR.

*Gene*	NCBI Seq. Ref.		Primers	bp
*BAX* [35]	XM_008252361.2	F	CCTTTTGCTTCAGGGTTTCA	165
		R	ATCCTCTGCAGCTCCATGTT	
*COX2* [50]	NM_001082388.1	F	CCTCACTGATGGGCTGTTTT	121
		R	GGTGAAAGCAATGCCTGAAT	
*IL1B* [35]	NM_001082201.1	F	TGAGGCCGATGGTCCCAATTA	183
		R	AAGGCCTGTGGGCAGGGAAC	
*TNFA*	NM_001082263.1	F	TCCTACGTGCTCCTCACTCA	170
		R	GGCGTCTTCCAGTTGGAGAA	
*18S* [51]	X03205.1	F	CGATCAGATACCGTCGTAGT	148
		R	TTCCTTTAAGTTTCAGCTTTGC	

BAX: BCL2-associated X protein; COX2: cyclooxygenase-2; IL1B: interleukin-1beta; TNFA: tumor necrosis factor-alpha; bp: base pair.

## Data Availability

The data presented in this study are available on request from the corresponding authors.

## Supplementary Materials

The following are available online at https://www.mdpi.com/article/10.3390/ani11061727/s1, Figure S1: Negative immunoreaction for BAX, COX2, IL1B and TNFA in rabbit ovaries. Figure S2: RT-qPCR standard curves with correlation coefficient and PCR efficiency for *BAX*, COX2, *IL1B*, *TNFA*, and *18S* primers. Figure S3: Immunohistochemical reactivity of primordial follicles for BAX and IL1B and of follicular cells in primary follicles for TNFA and COX2. Figure S4: Full immunoblots of BAX, COX2, and beta-tubulin proteins in the ovary of rabbits. Table S1. Ingredients and chemical composition of the commercial pelleted feed.
