# Supplementary material for: Effects of Dietary Polyphenols from Olive Mill Waste Waters on Inflammatory and Apoptotic Effectors in Rabbit Ovary
Source: Animals (Basel). 2021 Jun 9;11(6):1727. doi: 10.3390/ani11061727 (PMC8228552; doi:10.3390/ani11061727)
Supplement: Supplementary file 1 [file animals-11-01727-s001.zip › animals-1202091-supplementary.pdf]

animals

MDPI

Article

Effects of dietary polyphenols from olive mill waste waters on inflammatory and apoptotic effectors in rabbit ovary

Margherita Maranesi[1], Cecilia Dall'Aglio[1], Gabriele Acuti[1*], Katia Cappelli[1*], Massimo Trabalza-Marinucci[1], Roberta Galarini[2], Chiara Suvieri[3], Massimo Zerani[1]

[1] Dipartimento di Medicina Veterinaria, Università di Perugia, via San Costanzo 4, 06126, Perugia, Italy
[2] Istituto Zooprofilattico Sperimentale dell'Umbria e delle Marche "Togo Rosati", via Gaetano Salvemini 1, 06126, Perugia, Italy
[3] Dipartmento di Medicina e Chirurgia, Sezione di Farmacologia, piazzale Severi 1, 06132 Perugia, Italy.
* Correspondence: gabriele.acuti@unipg.it (G.A.); katia.cappelli@unipg.it (K.C.)

Table S1. Ingredients (% as fed basis) and chemical composition (g/100 g, unless otherwise indicated) of the commercial pelleted feed.

MATERIALS	CONCENTRATE[a]
Raw materials	
Dehydrated alfalfa meal	33.60
Wheat bran	16.85
Sunflower dehulled meal	11.50
Wheat flour middlings (tritello)	11.00
Barley grain flour	4.75
Sugar beet dried pulp	3.50
Carob germ	3.30
Sugar beet molasses	3.00
Corn grain flour	3.00
Grapeseed flour	2.20
Dehulled soybean grain flour	2.10
Wheat straw	2.00
Limestone	1.40
Vitamin-mineral premix[b]	1.00
Extruded linseed	0.50
Salt	0.30
Nutrients	
Crude protein	15.82
Crude fat	2.85
Ash	9.42
Neutral detergent fibre	38.19
Acid detergent fibre	22.22
Lignin (sa)[c]	6.18
Calcium	1.06
Phosphorous	0.54
Estimated digestible energy (MJ/kg)[d]	8.80

[a]Commercial pelleted concentrate. [b]Amount per kg of feed: Vitamin A 10.000 UI; Vitamin D3 800 UI; Vitamin E 40 mg; I 0.80 mg; Mn 28 mg; Se 0.16 mg; Zn 40 mg. [c]Lignin determined by solubilisation of cellulose with sulphuric acid. [d]According to Maertens *et al.* [40].

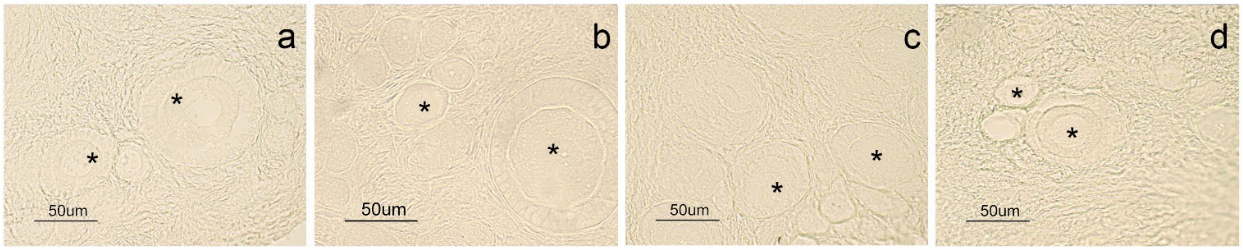

Figure S1. Pictures of rabbit ovaries in which no positive immunoreaction for BAX (a), COX2 (b), IL1B (c) and TNFA (d) was detectable. Asterisks indicate some follicles.

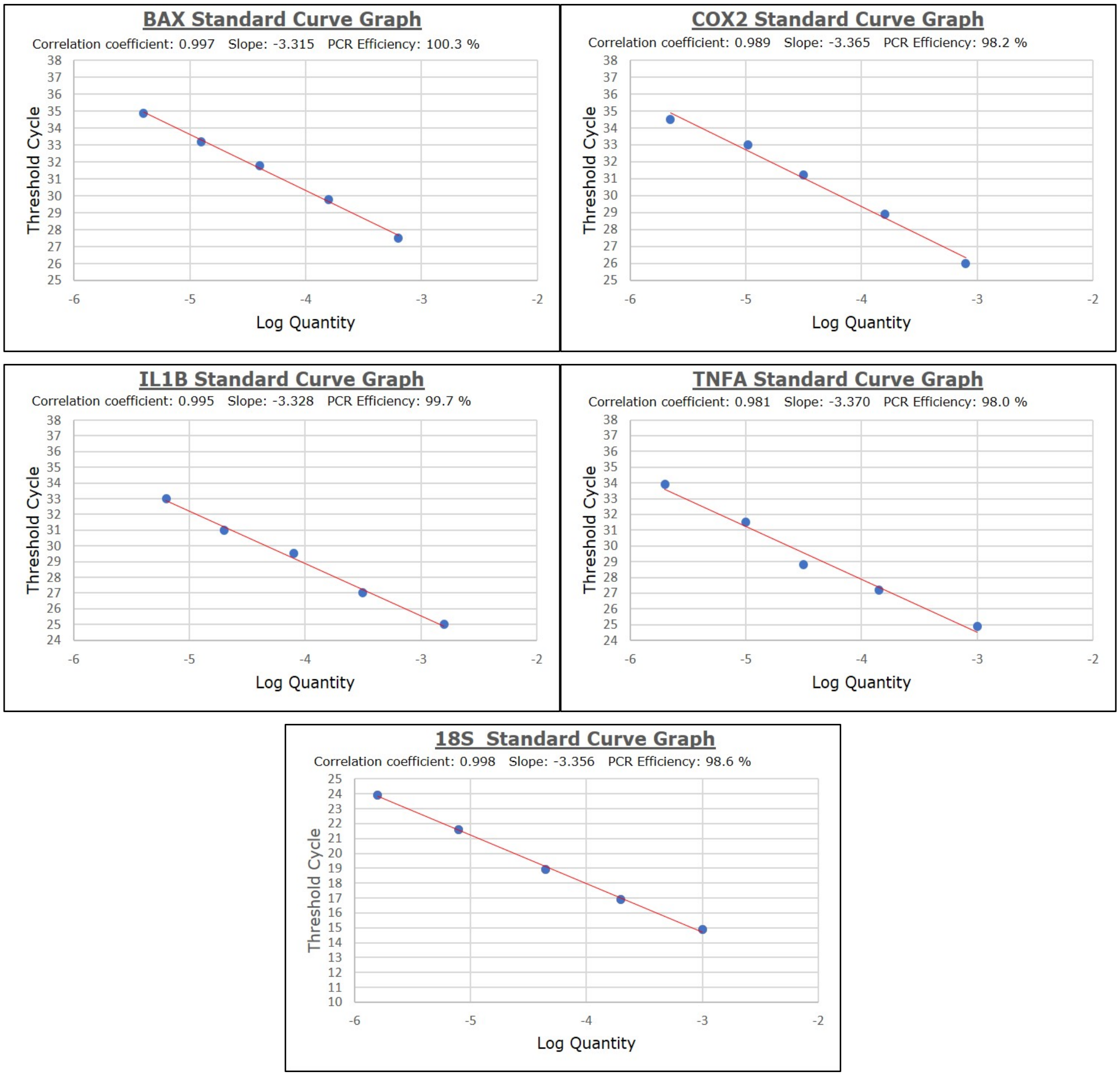

Figure S2. RT-qPCR standard curves with correlation coefficient and PCR efficiency for *BCL2-associated X protein (BAX), cyclooxygenase-2 (COX2), interleukin-1beta (IL1B), tumor necrosis factor-alpha (TNFA), and 18S* primers. Standard curves were generated by plotting the threshold value (threshold cycle, Ct) against the log cDNA standard dilution (1/5 dilution).

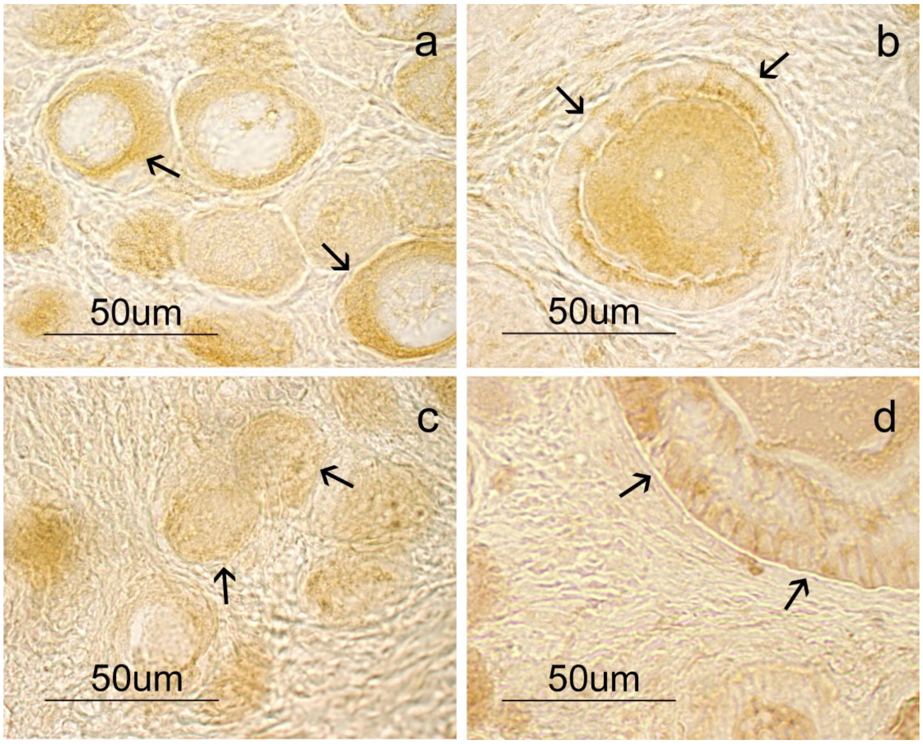

Figure S3. Immunohistochemical reactivity of primordial follicles for BAX and IL1B (a and c, arrows); immunohistochemical reactivity of follicular cells in primary follicles for TNFA and COX2 (b and d, arrows).

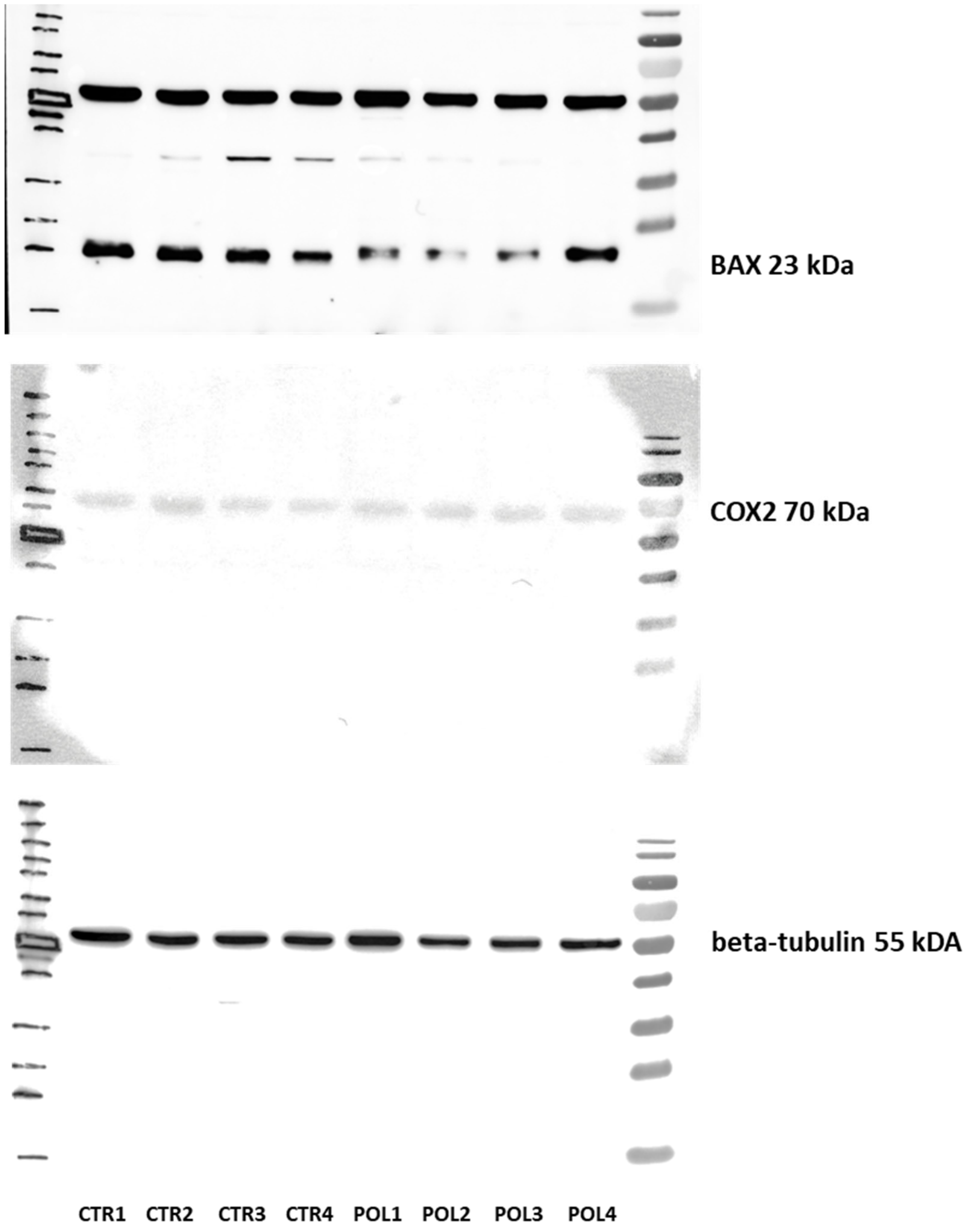

Figure S4. Full immunoblots of BAX, COX2, and beta-tubulin proteins in the ovary of rabbits
